# Sense of competence questionnaire among informal caregivers of older adults with dementia symptoms: A psychometric evaluation

**DOI:** 10.1186/1745-0179-3-11

**Published:** 2007-07-23

**Authors:** Aaltje PD Jansen, Hein PJ van Hout, Harm WJ van Marwijk, Giel Nijpels, Chad Gundy, Myrra JFJ Vernooij-Dassen, Henrica CW de Vet, François G Schellevis, Wim AB Stalman

**Affiliations:** 1 EMGO Institute, VU University medical center, Amsterdam, the Netherlands; 2 Department of General Practice, VU University medical center, Amsterdam, The Netherlands; 3 Leiden University Medical Center, department of Public health and Primary care, Leiden, The Netherlands; 4 Netherlands Cancer Institute-Antoni van Leeuwenhoek Hospital NKI-AvL, Department of Psycho Social Research and Epidemiology, Amsterdam, The Netherlands; 5 Alzheimer Centre UMC Nijmegen, the Netherlands; 6 Nivel, Netherlands Institute for Health Services Research, Utrecht, the Netherlands

## Abstract

**Background:**

The Sense of Competence Questionnaire (SCQ) was originally developed for informal caregivers of patients with diagnosed dementia. In order to study the validity and usefulness of the SCQ when applied to informal caregivers of older adults with dementia symptoms (i.e. cognitive impairment, pre-diagnostic dementia or dementia in its early stages), we investigated the construct validity, feasibility, subscales, homogeneity, and floor and ceiling effects in this new target population.

**Methods:**

A psychometric evaluation was performed among 99 informal caregivers. To investigate construct validity, hypotheses were tested, concerning the association between sense of competence and burden, mental quality of life, depressive symptoms, and mastery. To investigate feasibility, response rate and the proportion of missing data were explored for each item. An exploratory principal component analysis was used to investigate whether the SCQ comprises the three subscales established in previous studies. Homogeneity was assessed for each subscale with Cronbach's α and item-total correlations. Floor and ceiling effects were explored.

**Results:**

Most hypotheses on construct validity were rejected. Only the subscale 'consequences of involvement in care' was found to be partly valid. Feasibility: 93 out of 99 persons completed the SCQ. The proportion of unanswered items per item ranged from 0 – 3%. Subscales: the SCQ comprises the three expected subscales. Homogeneity: Cronbach's alpha and item-total correlations of the three subscales were satisfactory. A ceiling effect occurred on the subscale 'satisfaction with the care recipient'.

**Conclusion:**

The three subscales of the SCQ showed good homogeneity and feasibility, but their validity is insufficient: only the subscale 'consequences of involvement' was found to be partly valid. The two other subscales might not be relevant yet for the new target population, since many of the items on these scales refer to problem behaviour and problematic interactions. Our message to clinicians is not to use these subscales.

## Background

Even in its early stages, dementia may have a major impact on informal caregivers because of its chronic progressive and depersonalizing nature. Informal caregivers are persons who provide unpaid assistance to relatives and friends who have health problems or functional needs. They play an essential role in the provision of long-term care to community-dwelling older adults with cognitive impairment and with dementia [1,2]. Caregiving is generally unplanned and most informal caregivers gradually adopt their role because of the insidious nature of cognitive impairment and dementia [3]. Furthermore, caregiving may be a physically and emotionally demanding daily task that often lasts for years. The caregiving experience may provide emotional benefits to the caregiver, but it may also have adverse psychological, physical, social, and financial consequences [1,2].

Valid tools to measure the effects of care in informal caregivers of older adults with dementia symptoms (i.e. cognitive impairment, pre-diagnostic dementia or dementia in its early stages) are necessary. An important concept in the evaluation of effects of care is 'sense of competence'. This concept denotes the caregiver's feeling of being capable to care for the care recipient. The Sense of Competence Questionnaire (SCQ) measures this concept. The SCQ was originally developed for informal caregivers of patients with diagnosed dementia. It consists of three domains, identified by principal-components analysis in the original target population: 1. satisfaction with the care recipient, 2. satisfaction with one's own performance, and 3. consequences of involvement in care for the personal life of the caregiver. The SCQ has been validated among informal caregivers of older adults with diagnosed dementia and, later, in stroke caregivers. In both populations, it was found to be a valid instrument [4,5]. Content validity among informal caregivers of patients with diagnosed dementia was evaluated on the basis of classifications of the items made by a panel of experts, including professional caregivers and clinical researchers. The three dimensions of the SCQ were shown to have a high degree of correspondence with classifications made by this panel. Construct validity was checked with a principal-components analysis that revealed the three subscales [5].

However, the SCQ has never been used for informal caregivers of older adults with dementia symptoms. Therefore, we wanted to know whether the SCQ is a useful and valid questionnaire for this new target population. We gathered information on how this specific group performs on the SCQ because this may be different from informal caregivers of patients with diagnosed dementia. Informal caregivers of older adults with dementia symptoms may experience less distress due to behavioural problems of their care recipient than informal caregivers of patients with a diagnosis of dementia. Moreover, they may experience less adverse consequences of caregiving for their personal life. Therefore, we examined psychometric properties (construct validity, feasibility, subscales, homogeneity, and floor and ceiling effects) of the SCQ in informal caregivers of older adults with dementia symptoms.

## Methods

### Design

This study is a psychometric evaluation of the SCQ alongside a randomised clinical trial among primary informal caregivers of community-dwelling older adults with dementia symptoms. Baseline measurements of the trial were used. Caregivers entered the study after completing and returning an informed consent form. The Medical Ethics Committee of the VU University medical center in Amsterdam approved the study.

### Participants

99 pairs of informal caregivers and their care recipients participated in the trial. Care recipients were 65 years and older and lived at home in West-Friesland, a region in the northern part of the Netherlands. They received no assistance from outpatient geriatric services or outpatient diagnostic services and they had scores on the Mini Mental State Examination (MMSE) [6] below 24 or they had a risk of dementia of 50% or more according to the seven Minute Screen (7MS) [7]. Details on recruitment of participants have been described elsewhere [8]. In short, informal caregivers were recruited after screening for older adults with dementia symptoms in a large general practice population. Only primary informal caregivers were included. They were friends or relatives who were responsible for the informal care and who provided at least one hour of care a week. Exclusion criteria for patients were: terminal illness, insufficient command of the Dutch language, and participation in other research projects. Exclusion criteria for caregivers were: terminal illness and insufficient command of the Dutch language.

### Instruments

#### SCQ

The SCQ comprises 27 items that are rated on a 5-point scale: 1 'yes, completely agrees', 2 'yes, agrees', 3 'on the one hand agrees but on the other hand disagrees', 4 'no, disagrees', 5 'no, completely disagrees' [5]. When caregivers do not know an answer they can indicate this. The SCQ was found to consist of three subscales: 1. satisfaction with the care recipient (7 items; range 7–35; Cronbach's alpha = 0.55); 2. satisfaction with one's own performance as a caregiver (12 items; range 12–60; Cronbach's alpha = 0.63); and 3. consequences of involvement in care for the personal life of the caregiver (8 items; range 8–40; Cronbach's alpha = 0.50). Two items were recoded in the opposite direction and item-scores were summed subsequently. Higher scores indicate better sense of competence. Overall sum-scores were calculated in previous studies [5,9]. These scores ranged from 27–135. Next to sum-scores based on raw item-scores, sum-scores based on dichotomized item-scores (≤ 3 versus > 3) were calculated in previous research [5].

Apart from caregivers' sense of competence, the following caregiver variables were covered: age, gender, educational level, living situation, marital status, months spent on caring, hours spent on caring a week, help from other persons, time spent on caring a week, self-reported health, chronic diseases, level of caregiver's distress due to patient's behavioural problems measured with the distress scale of the Neuropsychiatric Inventory-Questionnaire (NPI-Q) [10], caregiver's burden measured with the Self-Perceived Pressure by Informal Care questionnaire (SPPIC) [11], caregiver's mental quality of life as determined with the mental component summary score of the MOS 36-item short-form health survey (SF-36) [12], mastery (i.e. the extent to which one regards one's life chances as being under one's own control in contrast to being fatalistically ruled) as measured with the Mastery scale [13], and, depressive symptoms measured with the Center for Epidemiologic Studies Depression Scale (CES-D) [14]. Furthermore, we collected the following care recipient characteristics: cognitive functioning measured with the Mini Mental State Examination (MMSE) [6], patients' initiative to perform self-care and patients' actual performance of self-care measured with the Interview for Deterioration in Daily life in Dementia (IDDD) [15], severity of behavioural problems measured with the severity scale of the Neuropsychiatric Inventory-Questionnaire (NPI-Q) [10], and, duration of cognitive problems in months.

Trained interviewers visited participating caregivers to obtain the SCQ and IDDD. Moreover, they picked up a caregiver-completed questionnaire. This postal questionnaire covered all remaining variables, described above, with the exception of cognitive functioning (MMSE). Cognitive functioning of care recipients was measured before baseline measurements of the trial.

To investigate construct validity, the SCQ was compared with measurements of caregiver's burden, caregiver's mental quality of life, depressive symptoms, and mastery. These measurements are described in more detail below.

#### SPPIC

The SPPIC is a 9-item self-report Rasch scale that measures self-perceived pressure from informal care. Items are scored on a 5-point scale: 1 'no!', 2 'no', 3 'more or less', 4 'yes', 5 'yes!'. To score the SPICC, item-scores are dichotomized and summed subsequently [11]. Scores 1 and 2 are recoded into 0 (i.e. not perceiving pressure) and scores 3, 4 and 5 are recoded into 1 (i.e. perceiving pressure). Scores range from 0 to 9 with higher scores indicating more pressure [11].

#### SF-36

The SF-36 is composed of 36 questions and standardized response choices, organized into eight multi-item scales. Besides, two summary scales, the Physical Component Summary (PCS) measure and the Mental Component Summary (MCS) measure can be calculated. Only the MCS is used for this study. Raw scale scores are linearly converted to a 0 to 100 scale, with higher scores indicating higher levels of functioning or well being [12].

#### CES-D

The CES-D is a 20-item self-report scale for assessing depressive symptoms. It asks subjects to describe how often they had depressive symptoms over the past week. Items are rated on a 4-point scale from 0 'rarely or none of the time to 3 'most or all of the time'. Scores range from 0 to 60, with scores over 15 indicating possible depression [14].

#### Mastery

The mastery scale is composed of 7 items. Items are rated on a 5 point scale: 1 'yes, completely agrees', 2 ' yes, agrees', 3 'on the one hand agrees but on the other hand disagrees', 4 'no, disagrees', 5 'no, completely disagrees'. Two items were recoded in the opposite direction. Subsequently, item-scores were summed and divided by the number of items. No missing items were allowed. Scores ranged from 7 to 35, with higher scores indicating better mastery [13].

### Analysis

#### Feasibility

Response rate and the percentage of missing values per item were calculated.

#### Subscales of the SCQ

First, we ran an exploratory principal component analysis (PCA) to check whether the SCQ measured the three domains established before [5]. As a consequence of the small sample size, performing a confirmatory analysis was not considered appropriate. We selected factors on the basis of the Scree test [16], i.e. we looked for a break between the factors with relatively large eigenvalues and those with smaller eigenvalues. Factors that appeared before the break were assumed to be potentially useful. Then, we conducted a forced three-factor analysis with oblique rotation (direct oblimin), similar to the study among informal caregivers of demented care recipients [5], to check and compare factor structure and loadings with those in the study among caregivers of demented care recipients.

#### Homogeneity

Homogeneity was assessed per subscale of the questionnaire. It was checked with Cronbach's α and the item-total correlations, both in raw and imputed data for which missing values were replaced with series means. Cronbach's α between 0.70 and 0.90 is considered to be adequate [17]. Items should correlate with the total score between 0.20 and 0.80 [17].

#### Floor and ceiling effects

We explored the presence of floor and ceiling effects by examining the frequency of highest and lowest possible scores at baseline SCQ-domain scores. Floor effects were considered present if more than 15% of participants had a minimal score at baseline, ceiling effect were considered present if more than 15% of participants had a maximum baseline score [18]. If ceiling or floor effects are present, a scale is unable to detect an improvement or decline in sense of competence in a considerable part of the target population.

#### Construct validity

Based on an underlying theory of what sense of competence is, one can hypothesize how the concept 'sense of competence' correlates with other concepts. If many of the hypotheses will be confirmed in the new target population, construct validity is good. We hypothesized a priori:

1. A moderate to strong negative association (r_s _= [-0.40, -0.80]) between caregivers' sense of competence and self-perceived burden. It is plausible that these two concepts influence each other because burden, referring to the consequences of the impaired person's restrictions for the caregiver, decreases the sense of competence referring to the caregiver's capability in caring for the impaired person [9].

2. A moderate to strong positive association (r_s _= [0.40, 0.80]) between caregivers' sense of competence and mental quality of life, because it is plausible that mental quality of life influences sense of competence and the other way around.

3. A moderate to strong negative association r_s _= [-0.40, -0.80] between caregivers' sense of competence and depressive symptoms, because it is plausible that depressive symptoms influences sense of competence and the other way around.

4. A moderate to strong positive association r_s _= [0.40, 0.80] between caregivers' sense of competence and mastery, because it is plausible that the extent to which one regards one's life chances as being under one's own control (i.e. sense of competence in general) influences sense of competence in caring, and the other way around.

We examined per subscale of the SCQ associations between the SCQ and caregiver's burden (SPPIC), caregiver's mental quality of life (MCS of the SF-36), caregiver's depression (CES-D) and mastery (Mastery) by calculating Pearson's correlation coefficients and their 95% confidence intervals. Correlations in the range 0.40 to 0.80 were regarded as moderate to strong associations [17]. Besides, we checked whether caregivers with low burden, with a high reported mental quality of life, without clinical relevant depressive symptoms and with a high reported mastery reported higher mean SCQ scores than the remaining caregivers. Therefore, we recoded burden, mental quality of life, depression and mastery scores in two ways: into three categories with equal distances and into quartiles, i.e. four categories with equal numbers of caregivers. Furthermore, we dichotomized CES-D scores into clinical relevant depressive symptoms (i.e. CES-D ≥ 16) and no clinical relevant depressive symptoms (i.e. CES-D < 16) [19].

## Results

Ninety-three informal caregivers out of 99 participating informal caregivers completed the SCQ. Five caregivers completed the postal questionnaire, but not the interview due to logistic problems. Furthermore, the research-team did not receive the SCQ and postal questionnaire of one caregiver due to problems with the Post Office. Table 1 presents the characteristics of the 93 participants who completed the SCQ, and their care recipients.

**Table 1:** Characteristics of participating caregivers and their care recipients (n = 93)

Characteristics	Value	N^a^
**Caregivers' socio-demographics**		
Age, mean ± SD (range)	62.9 ± 14.4 (32.5–91.2)	87
Gender, female (%)	71	93
Relation with the care recipient		92
Spouse	41%	
Child	50%	
Child in law	4%	
Other (friend, other member of the family)	5%	
Married (%)	83	86
Widowed (%)	1	
Living together with the care recipient (%)	47	93
Months spent on caring, median (25th percentile, 75th percentile)	24.0 (16.0, 48.0)	63
Hours spent on caring a week, median (25th percentile, 75th percentile)	7.0 (3.0, 41.0)	65
Help from other persons/shared care (%)	61	83
Educational level, primary school or no education (%)	15	88
**Caregivers' sense of competence**		
Overall SCQ, mean ± SD (range)	107.7 + 13.7 (65.9–132.0)	93
Subscale 1. Satisfaction with one's own performance as a caregiver, mean ± SD (range)	49.2 ± 6.5 (29.0–60.0)	93
Subscale 2. Consequences of involvement in care for the personal life of the caregiver, mean ± SD (range)	28.6 ± 6.2 (13.0–40.0)	93
Subscale 3. Satisfaction with the care recipient, mean ± SD (range)	29.9 ± 4.2 (16.0–35.0)	93
*Caregivers' general (health) functioning*		
Self reported health, good, very good or excellent health (%)	69	88
Chronic disease, one or more chronic diseases (%)	67	88
Caregiver's burden, SPPIC, mean ± SD (range)	3.5 ± 2.6 (0–9.0)	82
Mastery, mastery, mean ± SD (range)	25.3 ± 4.9 (16.0–35.0)	85
Depressive symptoms, CES-D, mean ± SD (range)	10.9 ± 6.9 (0–35.0)	88
Mental quality of life, MCS of the SF-36, mean ± SD (range)	49.5 ± 9.8 (23.6–68.1)	88
Caregiver's distress associated with patient's neuropsychiatric symptoms, NPI-Q distress, mean ± SD (range)	7.7 ± 8.3 (0–38.0)	84
*Patients*		
Cognitive functioning, MMSE-score, mean ± SD (range)	22.4 ± 4.0 (8–28)	90
Months with symptoms, median (25th percentile, 75th percentile)	26.0 (19.0, 48.0)	65
Severity of neuropsychiatric symptoms, NPI-Q, mean ± SD (range)	6.3 ± 5.6 (0–27.0)	84
Initiative to perform self-care, IDDD, mean ± SD (range)	11.9 ± 8.5 (0–32.0)	82
Actual performance of self-care, IDDD, mean ± SD (range)	13.7 ± 9.7 (0–40.0)	84

N^a ^= number of completed questionnaires; SD = Standard Deviation; SCQ = Sense of Competence Questionnaire; SPPIC = Self-Perceived Pressure of Informal Care questionnaire; CES-D = Center for Epidemiologic Studies Depression Scale; MCS = Mental Component Summary; SF-36 = MOS 36-item Short-Form health survey; NPI-Q = Neuropsychiatric Inventory-Questionnaire; MMSE = Mini Mental State Examination; IDDD = Interview for Deterioration in Daily life in Dementia

Participating informal caregivers of older adults with dementia symptoms reported better sense of competence than informal caregivers of older adults with diagnosed dementia (mean dichotomized score in dementia caregivers:17.9; sd: 5.2 [5]; mean dichotomized score in our participants: 21.3; sd: 4.4). Furthermore, our participants reported little distress associated with patients' behavioural problems, as well as low severity of behavioural problems in patients.

### Feasibility

A completed SCQ of 93 out of the 99 participating caregivers (94%) was received. Among those who completed the SCQ, the percentage of missing values per item ranged from 0% to 3%. On 18 items no missing values occurred.

### Subscales of the SCQ

**Figure 1:**
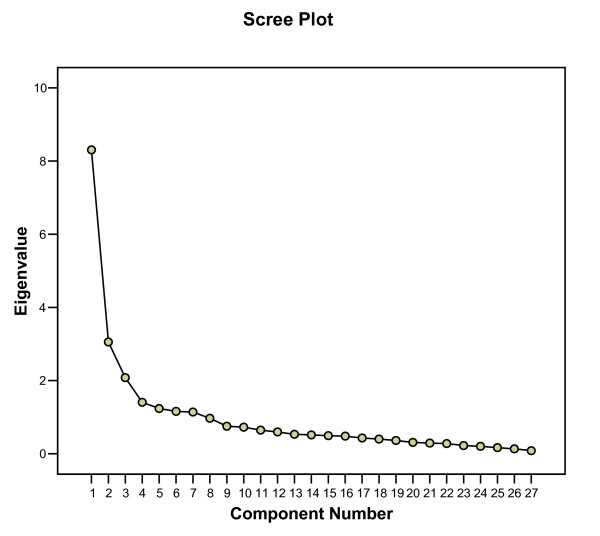
Scree plot of Eigenvalues of the SCQ.

Exploratory principal component analyses showed that the SCQ measured three distinct constructs, as was expected. The Scree plot in Figure 1 shows a distinct break before factor four, suggesting that only the first three factors were potentially useful enough to be retained. A forced three-factor analysis with an oblique rotation (direct oblimin) revealed that variables loading on the three factors were similar to those in the original questionnaire [5]. Factor loadings in our study population, as well as those in the original study among informal caregivers of patients with diagnosed dementia, are shown in Table 2. Items that loaded high on the first factor were those related to satisfaction with the care recipient. Moreover, items that loaded high on the second factor were related to consequences of involvement in care for the personal life of the caregiver, and items that loaded high on the third factor were related to satisfaction with one's own performance. However, only the items of the subscale 'consequences of involvement in care' all showed simple structure and they were associated well to the factors that they were grouped together with in the original questionnaire. Likewise, in the original questionnaire only the items of the subscale 'consequences of involvement in care' all showed simple structure. In our study, items 1, 2, 3, 5, 10, 11, 18, and 19 did not demonstrate simple structure, and the items 1,2, 10, 11, 18, and 19 were not associated well to the factors that they were grouped with in the original questionnaire.

**Table 2:** Principal Component Analysis: eigenvalues and factor loadings after oblique (direct oblimin) rotation.

Item	Factor 1. Satisfaction with the care recipient		Factor 2. Consequences of involvement in care for the personal life of the caregiver		Factor 3. Satisfaction with one's own performance	
*Satisfaction with one's own performance*	Our study	Original questionnaire	Our study	Original questionnaire	Our study	Original questionnaire
1. I feel pleased about my interactions with my ....	.44	.20	-.24	-.40	**.20**	-.79
2. I don't feel capable to care for my ....	-.04	-.09	.04	-.18	**.27**	.63
3. I wish that my .... And I had a better relationship.	.35	.06	-.07	.05	**.53**	.56
4. I feel guilty about my interactions with my ....	-.02	.01	-.15	-.10	**.68**	.50
5. I feel strained in my interactions with my ....	.38	.14	-.21	.29	**.48**	.50
6. I feel that in the past, I haven't done as much for my .... as I could have or should have.	-.07	-.08	.09	-.14	**.72**	.49
7. It is not clear to me how much care I should give to my ....	-.05	-.15	.10	-.00	**.74**	.40
8. I feel that my .... doesn't benefit from what I do for him/her.	.00	.08	-.04	-.03	**.77**	.37
9. I feel nervous or depressed about my interactions with my ....	-.03	.17	-.32	.30	**.44**	.34
10. I feel angry about my interactions with my ....	.39	.28	-.33	.04	**.33**	.32
11. I feel that I don't do as much for my .... as I should do.	.41	-.04	-.24	-.16	**.25**	.31
12. I feel useful in my interactions with my ....	.14	-.02	.12	-.04	**.32**	-.28
*Satisfaction with the care recipient*						
13. I feel that my .... behaves the way s/he does to have her/his own way.	**.82**	.89	-.06	.02	-.04	-.13
14. I feel that my .... behaves the way s/he does to annoy me.	**.83**	.72	.09	-.11	-.02	.04
15. I feel that may .... tries to manipulate me.	**.90**	.68	.13	.09	-.15	-.18
16. My .... appreciates my constant care less than the care others give him/her.	**.61**	.60	.05	-.06	.26	.20
17. I feel that my .... makes requests, which I perceive to be over and above what s/he needs.	**.50**	.59	-.39	.11	-.09	-.14
18. I feel resentful about my interactions with my ....	**.35**	.49	-.35	-.03	.35	.32
19. I feel embarrassed over my .... behaviour.	**.24**	.40	-.31	-.14	.37	.36
*Consequences of involvement in care*						
20. I feel that my present situation with my .... doesn't allow me as much privacy as I'd like.	.08	.01	**-.68**	.67	-.05	-.03
21. Because of my involvement with my .... I don't have enough time for myself.	-.02	-.00	**-.85**	.69	-.07	-.19
22. I feel that my social life has suffered because of my involvement with my ....	.13	-.02	**-.76**	.57	.04	.05
23. I feel that I cannot leave my .... alone, he/she needs me continuously.	.10	-.04	**-.70**	.52	-.08	-.08
24. I feel stressed between trying to give up my .... as well as to other family responsibilities, job etc.	-.03	.03	**-.63**	.49	-.05	.18
25. I feel that my health has suffered because of my involvement with my ....	-.06	-0.03	**-.66**	.46	.19	.20
26. I worry all the time about my ....	-.30	-0.29	**-.71**	.44	.06	.27
27. I feel that my .... Seems to expect me to take care of him/her as if I were only one s/he could depend on.	.12	.17	**-.66**	.37	-.16	-.16

Unrotated:						
Eigenvalue	8.39	-	3.03	-	2.02	-
Variance explained	31%	-	11%	-	7%	-

Furthermore, factor 1 correlated weakly with factor 2 and 3 (0.28 and 0.32, respectively). Factor 2 correlated weakly with factor 3 (0.20).

### Homogeneity

The following figures on homogeneity were found on the subscales:

- satisfaction with the care recipient: Cronbach's α = 0.83 and range of item-total correlations: 0.50–0.68;

- satisfaction with one's own performance as a caregiver: Cronbach's α = 0.83 and range of item-total correlations: 0.16–0.70;

- consequences of involvement in care for the personal life of the caregiver: Cronbach's α = 0.85 and range of item-total correlations: 0.50–0.76.

The results were similar for imputed and raw data.

### Floor and ceiling effects

Floor and ceiling effects were not present with the exception of the subscale 'satisfaction with the care recipient'. Here, a ceiling effect occurred: 18% of the participants had a maximum score.

### Construct validity

The hypothesized outcomes and realized outcomes of the 4 hypotheses are summarized per subscale of the SCQ: 'satisfaction with one's own performance as a caregiver' in Table 3, 'consequences of involvement in care' in Table 4, and 'satisfaction with the care recipient' in Table 5. On the subscales 'satisfaction with one's own performance' and 'satisfaction with the care recipient' none of the four hypotheses was accepted. Only on the subscale 'consequences of involvement in care' the expected associations with burden and mental quality of life were found, but not with depression and mastery.

**Table 3:** Tested hypotheses on construct validity: Satisfaction with one's own performance as a caregiver

We expected:	Value outcome found	Hypothesis accepted?*
*Burden*		
A moderate to strong negative association with measures of caregivers' burden.	1. Association with caregivers' burden: r = -0.21, 95% Confidence Interval (CI): [-0.43, 0.01]; n = 82.	-
	2. Categories of burden: -equal distances: 49.5 (SPPIC 0–3, n = 45), 48.6 (3–6, n = 25), to 46.7 (6–9, n = 12); -equal observations: 49.9 (1, n = 21), 49.4 (2, n = 18), 49.6 (3, n = 23), 46.2 (4, n = 20).	-
*Mental quality of life*		
A moderate to strong positive association with measures of caregivers' mental quality of life.	1. Association with caregivers' mental quality of life: r = 0.24, 95% CI: [0.03, 0.46]; n = 88.	-
	2. Categories of mental quality of life: -equal distances: 46.1 (MCS 0–33.3, n = 8), 48.3 (33.3–49.9, n = 27), to 50.0 (49.9–100.0, n = 53); F = 1.535, p = 0.221; -equal observations: 47.3 (1, n = 22), 48.2 (2, n = 22), 50.1 (3, n = 22), 50.8 (4, n = 22); F = 1.434, p = 0.239.	-
*Depressive symptoms*		
A moderate to strong negative association with depressive symptoms.	1. Association with caregivers' depressive symptoms: r = -0.21, 95% CI: [-0.42, 0.004]; n = 88.	-
	2. Categories of depressive symptoms: -dichotomised: 47.4 (CES-D ≥ 16.0, n = 18), 49.5 (CES-D < 16, n = 70), student's t-test: p = 0.212.	-
*Mastery*		
A moderate to strong positive association with mastery.	1. Association with caregivers' mastery: r = 0.19, 95% CI: [-0.02, 0.41]; n = 85.	-
	2. Categories of mastery: -equal distances: 46.8 (mastery 16–21, n = 18), 49.3 (mastery 21–28, n = 45), to 50.7 (mastery 28–35, n = 21); -equal observations: 47.2 (1, n = 23), 50.5 (2, n = 20), 48.3 (3, n = 20), 50.7 (4, n = 22).	-

* hypothesis accepted:

+ accepted

- rejected

**Table 4:** Tested hypotheses on construct validity: Consequences of involvement in care

We expected:	Value outcome found	Hypothesis accepted?*
*Burden*		
A moderate to strong negative association with measures of caregivers' burden.	1. Association with caregivers' burden: r = -0.69, 95% Confidence Interval (CI): -[1.00, 0.62]; n = 82.	+
	2. Categories of burden: -equal distances: 31.4 (SPPIC 0–3, n = 45), 26.7 (SPPIC 3–6, n = 25), to 19.4 (SPPIC 6–9, n = 12); F = 38.850, p < 0.001; -equal observations: 32.0 (1, n = 21), 31.0 (2, n = 18), 28.8 (3, n = 23), 21.1 (4, n = 20); F = 24.452, p < 0.001.	+
*Mental quality of life*		
A moderate to strong positive association with measures of caregivers' mental quality of life.	1. Association with caregivers' depressive symptoms: r = 0.44, 95% CI: [0.14, 0.57]; n = 88	+
	2. Categories of mental quality of life: -equal distances: 23.5 (MCS 0–33.3, n = 8), 26.0 (MCS 33.3–49.9, n = 27), to 30.1 (MCS 49.9–100.0, n = 53); F = 7.615, p = 0.001; -equal observations: 24.6 (1, n = 22), 28.1 (2, n = 22), 28.1 (3, n = 22), 32.2 (4, n = 22); F = 6.462, p = 0.001.	+
*Depressive symptoms*		
A moderate to strong negative association with depressive symptoms.	1. Association with caregivers' depressive symptoms: r = -0.27, 95% CI: -[0.49, -0.06]; n = 88	-
	2. Categories of depressive symptoms: -dichotomised: 27.7 (CES-D ≥ 16.0, n = 18), 28.3 (CES-D < 16, n = 68), student's t-test: p = 0.716.	-
*Mastery*		
A moderate to strong positive association with mastery.	1. Association with caregivers' mastery: r = 0.34, 95% CI: [0.14, 0.57]; n = 85	-
	2. Categories of mastery: -equal distances: 24.9 (mastery 16–21, n = 18), 28.5 (mastery 21–28, n = 45), to 30.9 (mastery 28–35, n = 21); -equal observations: 25.9 (1, n = 23), 28.5 (2, n = 21), 28.2 (3, n = 19), 30.9 (4, n = 21).	-

* hypothesis accepted:

+ accepted

- rejected

**Table 5:** Tested hypotheses on construct validity: Satisfaction with the care recipient

We expected:	Value outcome found	Hypothesis accepted?*
*Burden*		
A moderate to strong negative association with measures of caregivers' burden.	1. Association with caregivers' burden: r = -0.23, 95% CI: [-0.46, -0.02]; n = 82.	-
	2. Categories of burden: -equal distances: 30.4 (SPPIC 0–3, n = 45), 30.2 (3–6, n = 25), to 27.1(6–9, n = 12); -equal observations: 30.7 (1, n = 21), 30.1 (2, n = 18), 30.7 (3, n = 23), 27.9 (4, n = 20).	-
*Mental quality of life*		
A moderate to strong positive association with measures of caregivers' mental quality of life.	1. Association with caregivers' mental quality of life: r = 0.16, 95% CI: [-0.05, 0.38]; n = 88	-
	2. Categories of mental quality of life: -equal distances: 29.4 (MCS 0–33.3, n = 8), 29.0 (33.3–49.9, n = 27), to 30.3 (49.9–100.0, n = 53); -equal distances: 28.4 (1, n = 22), 30.3 (2, n = 22), 30.0 (3, n = 22), 30.7 (4, n = 22).	-
*Depressive symptoms*		
A moderate to strong negative association with depressive symptoms.	1. Association with caregivers' depressive symptoms: r = -0.05, 95% CI: [-0.26, 0.16]; n = 88.	-
	2. Categories of depressive symptoms: -dichotomised: 30.0 (CES-D ≥ 16.0, n = 18), 29.8 (CES-D < 16, n = 70), student's t-test: p = 0.830.	-
*Mastery*		
A moderate to strong positive association with mastery.	1. Association with caregivers' mastery: r = 0.15, 95% CI: [-0.06, 0.37]; n = 85	-
	2. Categories of mastery: -equal distances: 28.9 (mastery 16–21, n = 18), 29.8 (mastery 21–28, n = 45), to 30.7 (mastery 28–35, n = 22); -equal observations: 28.9 (1, n = 23), 30.2 (2, n = 20), 29.8 (3, n = 20), 30.7 (4, n = 22).	-

* hypothesis accepted:

+ accepted

- rejected

## Discussion

The SCQ has been used for informal caregivers of older adults with diagnosed dementia, but has never been used for informal caregivers of older adults with dementia symptoms. This new target population performed differently on the SCQ than informal caregivers of patients with diagnosed dementia.

Unsurprisingly, participating informal caregivers of older adults with dementia symptoms reported better sense of competence than informal caregivers of older adults with diagnosed dementia.

### Feasibility

Feasibility was satisfactory as the proportion of unanswered items on the SCQ was very low.

### Subscales of the SCQ

Exploratory principal component analyses showed that the SCQ measured three constructs similar to those found in the study among caregivers of older adults with dementia [5]. However, only the items of the subscale 'consequences of involvement in care' all showed simple structure, just as on the original questionnaire.

### Homogeneity

Cronbach's alphas of the three subscales satisfied and were more adequate than those found in the source population in which the SCQ was validated [5].

### Floor and ceiling effects

Floor effects were absent. However, on the subscale 'satisfaction with the care recipient ' a ceiling effect occurred. This means that it is impossible to detect an improvement on this subscale in a considerable part of the target population. Furthermore, the subscale 'satisfaction with the care recipient' seems to be less relevant for our study population. The reason may be that the items of this subscale refer to problem behaviour. Probably, caregivers of persons with dementia symptoms are not yet familiar or do not encounter problems with problem behaviour, since participants reported low distress associated with patients' behavioural problems, as well as low severity of behavioural problems in patients.

### Construct validity

Most hypotheses were rejected. Only the subscale 'consequences of involvement in care for the personal life of the caregiver' was found to be partly valid. However, we do not know how the SCQ performs with regard to comparison questionnaires among informal caregivers of patients with diagnosed dementia, because no previous research has focused on this subject and in the original questionnaire, construct validity was determined by means of a principal component analysis.

The strength of this study is that we were able to compare sense of competence with several other related constructs in a new target population. However, this study has some limitations.

Firstly, comparison questionnaires were chosen based on the overall construct sense of competence. Our perception of this construct equalled the subscale 'consequences of involvement in care', but corresponded less well with the two other subscales 'satisfaction with one's own performance' and 'satisfaction with the care recipient'. However, that only partly explains the weakness of the correlations with the comparison questionnaires and these subscales. A more important explanation for the weak correlations might be that the two subscales are not very relevant yet for the new target population, since many items on these scales refer to problem behaviour and problematic interactions. Another explanation might be that the constructs are not related in the way we think "plausible".

Secondly, the study population may not be representative for all informal caregivers of older adults with cognitive impairment and dementia in its early stages, since the study population was recruited after screening for older adults with dementia symptoms in a large general practice population. Informal caregivers of non-respondents to the screening were not recruited, while these non-respondents were found to have higher rates of functional and cognitive impairment in other studies [20,21]. Thus, informal caregivers of more severely impaired older adults with dementia symptoms may be under-represented.

Thirdly, the comparison questionnaires used in examining the construct validity suffered from missing values on the sum-scores of the SPPIC, CES-D and SF-36. However, the influence of this small number of missing values on construct validity is limited as there is no reason to assume that the persons with missing values differed from the persons without such values since missing values were at random.

We recommend further research on the responsiveness to change and reproducibility (test-retest reliability) of the SCQ. Responsiveness to change can be investigated by relating the smallest detectable change, which is based on the standard error of measurement, to the minimal important change. The minimal important change can be estimated by relating changes in scores between two measurements to an external criterion. Furthermore, we recommend further investigation into the content validity of the SCQ by asking informal caregivers' opinion about the content of the items.

## Conclusion

In conclusion, among informal caregivers of older adults with dementia symptoms, the subscales of the SCQ showed good homogeneity and feasibility, but their validity is insufficient: only the subscale 'consequences of involvement in care for the personal life of the caregiver' was found to be partly valid. The two other subscales are not yet very relevant for the new target population, since many of the items on these scales refer to problem behaviour and problematic interactions while participants reported low distress associated with patients' behavioural problems, as well as low severity of behavioural problems in patients. Our message to clinicians is not to use the subscales 'satisfaction with one's own performance' and 'satisfaction with the care recipient' in informal caregivers of older adults with dementia symptoms who do not have behavioural problems or problematic interactions with their caregiver. Furthermore, the subscale 'satisfaction with the care recipient' is unable to detect an improvement in a considerable part of informal caregivers of older adults with dementia symptoms. Therefore, we advise caution when using the subscale 'satisfaction with the care recipient' to detect changes in levels of functioning among informal caregivers of persons with dementia symptoms.

## Competing interests

The author(s) declare that they have no competing interests.

## Authors' contributions

All authors have critically read and approved the final version of the manuscript.

DJ: responsible for the inclusion of participants, data-collection, analyses, interpretation of data, preparation of this manuscript

HvH: supervised the project

HvM: supervised the project

GN: supervised the project

CG: provided advice on statistically methods

RdV: provided advice on clinimetrics, contributed substantially to the interpretation of data

MD: provided advice on application of the SCQ

WS: supervised the project
